# Molecularly imprinted polymers outperform lectin counterparts and enable more precise cancer diagnosis†

**DOI:** 10.1039/d2sc01093c

**Published:** 2022-03-17

**Authors:** Jilei Pang, Pengfei Li, Hui He, Shuxin Xu, Zhen Liu

**Affiliations:** State Key Laboratory of Analytical Chemistry for Life Science, School of Chemistry and Chemical Engineering, Nanjing University 163 Xianlin Avenue Nanjing 210023 China zhenliu@nju.edu.cn +86-25-8968-5639

## Abstract

Accurately analysing the particular glycosylation status of protein biomarkers is of significant importance in the precise, early diagnosis of cancer. Existing methods mainly rely on the use of antibodies and lectins. However, due to the macroscopic and microscopic heterogeneity of glycans, precise analysis of glycosylation status still remains a challenge. Molecularly imprinted polymers (MIPs), as a synthetic alternative to antibodies or lectins, may provide new solutions but have not yet been explored. Herein, we report an appealing strategy called triple MIP-based plasmonic immunosandwich assay (triMIP-PISA) for precise cancer diagnosis in terms of the relative glycosylation expression of glycoprotein biomarkers. As proof of the principle, alpha fetoprotein (AFP), which has been used as a clinical biomarker for early detection of hepatocellular carcinoma (HCC), as well as its *Lens culinaris* agglutinin (LCA)-reactive fraction (AFP-L3), which is mainly composed of core-fucosylated glycans, were used as two target proteoforms to test in this study. Using two MIPs that can specifically recognize the peptide sequence of AFP as well as a fucose-imprinted MIP that can specifically recognize the AFP-L3 fraction, facile simultaneous plasmon-enhanced Raman detection of AFP and AFP-L3 in serum was achieved, which allowed HCC patients to be distinguished from healthy individuals. Due to the excellent recognition properties of the MIPs that are comparable to those of antibodies and superior to those of lectins, our triMIP-PISA method exhibited improved precision as compared with an antibody plus lectin-based immunofluorescence assay. Thus, this strategy opened a new avenue towards the precise diagnosis of cancer.

## Abstract 2

A triple molecularly imprinted polymer (MIP)-based plasmonic assay was developed for precise cancer diagnosis in terms of the relative glycosylation expression of glycoprotein biomarkers.

## Graphical abstract

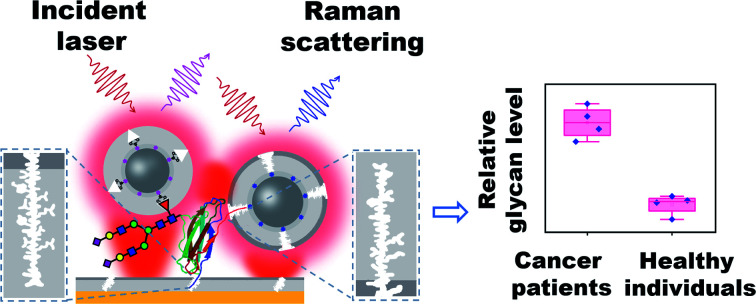

## Introduction

Glycosylation is a widely occurring post-translational modification of proteins. Glycosylated proteins play essential roles in important physiological processes in organisms, such as intercellular communication,^1–3^ signal transduction,^4–6^ and immune response.^7,8^ The expression of aberrant glycans is intimately associated with pathological conditions, including cancer,^9^ possibly due to dysregulated transcription of enzymes of the glycosylation machinery. Quite a lot of glycoproteins have been routinely used as disease biomarkers for early clinical diagnosis of diseases, especially cancer.^10,11^ Precise diagnosis of cancer at an early stage is of significant importance.^12–14^ Therefore, analytical methods that can accurately analyse the glycosylation status of protein biomarkers are in high demand in precise diagnosis.

At present, precise analysis of the glycosylation status of glycoproteins with important biological significance still faces huge challenges because of the macroscopic and microscopic heterogeneity of glycans.^15,16^ Methods permitting analysis of disease biomarker glycoproteins and their associated glycans mainly rely on mass spectrometry (MS),^17^ immunoassays,^18^ and lectin-based assays.^19,20^ MS has been the most prominent methodology for identifying sugar-containing biological molecules, including glycoproteins, glycopeptides and glycans. However, its application is often hampered by the poor ionization efficiency of sialic acid-containing glycans and inadequate resolving capability towards glycan isomers.^17^ Although antibodies have been employed to construct immunoassays of glycoproteins and their glycosylation, the number of glycan-specific antibodies is still limited. Lectins are unique biomolecules that are capable of recognizing specific glycan structures. Lectins have been widely used to analyse glycoconjugate structures in proteomics,^21^ glycomics,^22,23^ and diagnostics.^24,25^ However, lectins suffer from several disadvantages, including inadequate specificity and weak affinity. The dissociation constant (*K*_d_) of many lectins towards monosaccharides or oligosaccharides ranges from 10^−3^ to 10^−4^ M.^26–28^ Such weak affinity disables lectins as capture ligands to extract trace target species from complex clinical samples. To this end, the antibody-lectin sandwich array (ALSA) has been developed as an important alternative to immunoassays in clinical diagnosis.^29–31^ However, IgG, the most common antibody, has a pair of N-linked glycans in the constant region of the heavy chain.^32^ When lectins are used to bind target glycoproteins captured by antibodies, their binding with the antibodies may result in severe and uncontrolled blank readout.^33,34^ These disadvantages of antibodies and lectins severely hinder their application in accurate clinical diagnosis. Moreover, although aptamers, which are short single-stranded DNA or RNA or peptides with the ability to bind specific species, have been widely used as antibody alternatives to create immunoassays,^35–38^ aptamers capable of recognizing specific glycan structures of proteins also remain rare.^39^ Therefore, it is necessary to develop new solutions to these hurdles.

Molecularly imprinted polymers (MIPs), which are chemically synthesized in the presence of a template, exhibit affinity towards the template.^40–45^ As compared with antibodies, MIPs have several advantages, including ease of preparation, low cost, and good stability. In recent years, our group has developed several versatile and efficient imprinting methods that allow for facile preparation of MIPs specific to sugar-containing biological molecules, including glycoproteins,^46^ glycans,^47^ and monosaccharides.^48^ The prepared MIPs have enabled multiple promising applications, such as disease diagnosis,^49,50^ cell/tissue imaging,^51,52^ cancer therapy^53^ and smart prodrug delivery.^54^ By combining MIPs with plasmon-enhanced Raman scattering (PERS), which is an ultrasensitive detection method, we have developed an antibody- and enzyme-free approach called plasmonic immunosandwich assay (PISA).^55–57^ PISA has exhibited great potential to be a powerful tool for disease diagnosis.^58^ However, the unique strengths of MIPs and PISA in precise diagnosis have not been explored to date.

**Scheme 1:**
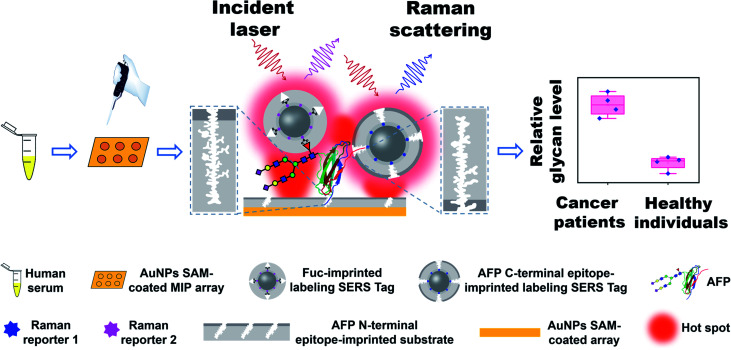
Schematic of the precise diagnosis of HCC *via* tri-molecularly imprinted polymer-based plasmonic immunosandwich assay.

Herein, we developed a new strategy called triple MIP-based PISA (triMIP-PISA) for precise disease diagnosis in terms of the relative glycosylation expression of glycoprotein disease biomarkers. This method benefits greatly from the excellent protein-recognizing properties of MIPs that approach those of monoclonal antibodies, as well as the excellent monosaccharide-recognizing properties of MIPs, outperforming lectins. As a proof of the principle, alpha fetoprotein (AFP), which has been a routinely used biomarker for the early screening of hepatocellular carcinoma (HCC), was used as the test glycoprotein biomarker in this study. AFP is an N-linked glycoprotein with a single glycosylation site, but the glycans are highly diverse (Fig. S1†). The relative expression level of the glycoform family L3 of AFP, which is mainly composed of core-fucosylated glycans, has been proven to be a malignant tumor-specific biomarker^59^ and is approved as a disease marker by the Food and Drug Administration (FDA) for early diagnosis of liver cancer.^60^ Currently existing analytical tools for the determination of the relative AFP-L3 level mainly rely on the use of antibodies and lectins.^61^ Due to their drawbacks as stated above, the precision of these assays remains not very high. To solve this issue, our new strategy turns to triple recognition of the glycoprotein biomarker by three different types of MIPs, integrated with plasmonic detection. The principle and procedure of triMIP-PISA are illustrated in Scheme 1. An N-terminal epitope-imprinted substrate was used to specifically extract the target glycoprotein from clinical samples, then the captured target molecules were labelled with C-terminal epitope-imprinted nanotags encapsulated with Raman reporter 1, while the fucosylated glycans of the glycoprotein were labelled with fucose (Fuc)-imprinted nanotags encapsulated with Raman reporter 2, which has characteristic Raman peaks distinct from those of reporter 1. Sandwich-like immunocomplexes formed on the substrate were subject to plasmonic detection. Raman signal generated by reporter 1-containing nanotags reports the total AFP level, while that by the reporter 2-containing nanotags reports the level of fucosylated glycans of AFP (also referred to as L3). Thus, the relative expression level of fucosylated glycoforms over the total level of AFP in human serum (AFP-L3/AFP) can function as a reliable and specific indicator for HCC patients. As a key to the precise diagnosis, Fuc-imprinted MIP was experimentally proven to apparently outperform a typical lectin that can recognize Fuc (LCA) in terms of specificity and affinity. Using the established method, facile simultaneous detection of AFP and AFP-L3 in serum was achieved, which distinguished HCC patients from healthy individuals. In comparison with the clinical detection method of ALSA for AFP and AFP-L3, the diagnostic accuracy based on the triMIP-PISA method has been apparently improved. Therefore, the triMIP-PISA opened a new avenue towards the precise diagnosis of diseases.

## Results and discussion

### Preparation of MIPs for constructing triMIP-PISA

**Fig. 1:**
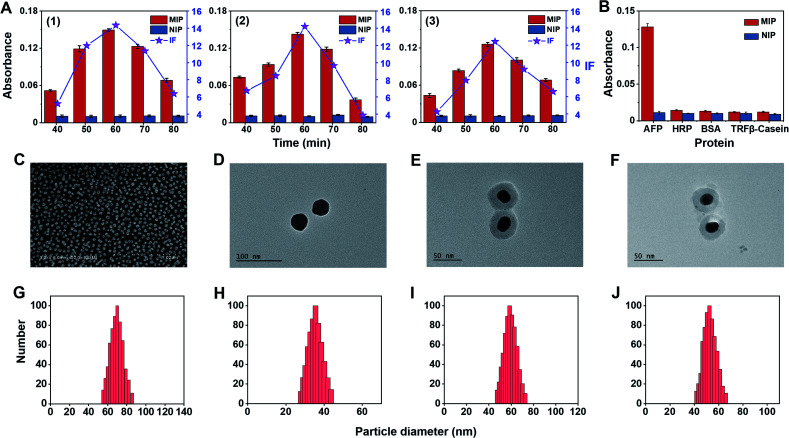
(A) Conditional optimization for the imprinting of AFP C-terminal epitope and selectivity test of the prepared MIP. Dependence of the amount of the AFP C-epitope peptide captured by imprinted and non-imprinted MNPs prepared with the molar ratio of APTES/UPTES/IBTES/TEOS at (1) 10 : 20 : 40 : 30, (2) 20 : 20 : 30 : 30, and (3) 10 : 20 : 50 : 20. (B) Comparison of the amounts of different proteins captured by AFP C-epitope-imprinted MNPs. The error bars represent the standard deviation for three parallel experiments. (C) SEM characterization of an N-terminal epitope-imprinted AuNP SAM coated-glass substrate. TEM images of (D) AgNPs, (E) C-terminal epitope-imprinted Ag/PATP@SiO_2_ NPs, and (F) Fuc-imprinted Ag/NTP@SiO_2_ NPs. DLS characterization of (G) AuNPs, (H) AgNPs, (I) C-terminal epitope-imprinted Ag/PATP@SiO_2_ NPs, and (J) Fuc-imprinted Ag/NTP@SiO_2_ NPs.

To construct triMIP-PISA, a MIP array specific to the N-terminal epitope of AFP was prepared on a AuNP self-assembled monolayer (SAM)-coated glass slide, while a MIP against the C-terminal epitope of AFP was fabricated onto Raman-active Ag-core nanoparticles (NPs) according to a new method called boronate affinity-anchored epitope oriented surface imprinting and cladding^62^ that we developed recently. As compared with its earlier version, *i.e.*, boronate affinity-assisted oriented surface imprinting,^63^ the new imprinting approach introduced an additional process called cladding, which can effectively improve the binding affinity and specificity. The *K*_d_ value of the AFP N-terminal epitope imprinted substrate could reach 10^−9^ M level, while its cross-reactivity toward interfering proteins was found to be ≤ 7.6%.^62^ Such excellent binding properties well ensured specific extraction of trace target protein from a complex sample matrix, such as serum. On the other hand, Fuc-imprinted Raman-active AgNPs were prepared according to the boronate affinity controllable-oriented surface imprinting^64^ we developed previously. The structures of the N-(RTLHRNEYGIAS) and C-terminal epitope (KLISKTRAALGV) of AFP are shown in Fig. S2,† and the glycated N-terminal epitope (Fru-RTLHRNEYGIAS) and glycated C-terminal epitope (KLISKTRAALGVK-Fru) of AFP were used as the imprinting templates. The synthesis routes of AFP N-terminal epitope-imprinted arrays, AFP C-terminal epitope-imprinted nanotags and Fuc-imprinted nanotags are shown in Fig. S3–S5,† and the details of the preparation procedures are given in the ESI.† Since the imprinting conditions for AFP N-terminal epitope-imprinted MIP and Fuc-imprinted MIP have been well optimized previously,^62,64^ no conditional optimization was carried out for these two types of MIPs. For C-terminal epitope-imprinted MIP, the monomer ratio and imprinting time were optimized, with the cladding time kept constant at 10 min as in a previous study,^62^ since it is not the major factor that determines the binding properties. Magnetic nanoparticles (MNPs) were chosen as a substrate material for the convenience of optimization, because of easy magnetic separation. First, boronic acid-functionalized Fe_3_O_4_@SiO_2_ MNPs were prepared. Then, glycated C-terminal epitope of AFP was immobilized onto the prepared MNPs for boronate affinity-based oriented surface imprinting. According to the features of amino acids of the epitope sequence, as shown in Fig. S2B,† 3-aminopropyltriethoxysilane (APTES), 3-ureidopropyltriethoxy-silane (UPTES), isobutyltrieth-oxysilane (IBTES), and tetraethyl-orthosilicate (TEOS) were selected as functional monomers to interact with the epitope peptide *via* electrostatic, hydrogen bonding, and hydrophobic interactions, respectively, while TEOS was used as a cross-linker to form a hydrophilic silica skeleton. After optimization of the ratio of monomers and cross-linker, the thickness of the imprinting layer was controlled by imprinting time. Imprinting factor (IF), which is the ratio of the amount of epitope captured by imprinted MNPs and the non-imprinted MNPs, was used to evaluate the imprinting effect. The optimization results for C-terminal epitope-imprinted MNPs are shown in Fig. 1A. The imprinting with the APTES/UPTES/IBTES/TEOS ratio at 10 : 20 : 40 : 30 for 60 min exhibited the best IF value (14.4), which is outstanding in epitope imprinting. The specificity of C-epitope-imprinted MNPs prepared at the optimal imprinting conditions was investigated. The results show that the C-epitope-imprinted MNPs exhibited excellent specificity, yielding cross-reactivities less than 11.2% toward non-target proteins, including HRP, BSA, transferrin (TRF) and β-casein (Fig. 1B).

Because the imprinting on silver nanoparticles involved several steps, the optical stability of MIPs during the preparation process was investigated. Fig. S6A† shows the UV-vis spectra at different steps. It was found that the UV-vis spectra was redshifted after being modified with Raman reporters (1 and 2) and boronic acid. The reason is that the Raman reporters and boronic acid have electron-withdrawing groups, which could cause the redshift of the spectra. The redshift of the UV-vis spectra also convincingly indicates that the modification of Raman reporters and boronic acid were successful. Meanwhile, the spectral profiles of these steps for imprinted and non-imprinted materials are consistent with each other, suggesting good optical stability of these particles in the preparation process. To confirm the stability of the Raman signal of MIPs during the preparation process, the changes in Raman response for silica coating and imprinting were investigated. As shown in Fig. S6B,† there were no obvious changes in the Raman spectra before and after silica coating and imprinting. The peak at 1435 cm^−1^, a characteristic peak of 4-aminothiophenol (PATP), was selected as a quantitative indicator for total AFP, while the characteristic peak of 4-nitrothiophenol (NTP) at 1330 cm^−1^ was selected as a quantitative indicator for AFP-L3. The two selected characteristic peaks had no overlap, which allowed for simultaneous quantitative measurement of total AFP and AFP-L3.

To make a plasmonic substrate that can enhance the Raman signal intensity of nanotags located on it, gold nanoparticles (AuNPs) were prepared. All nanomaterials involved in this study were characterized. Fig. 1C–J show the SEM and TEM images and DLS results. As characterized by SEM and DLS, AuNPs were found to be uniform and monodispersed on the substrate, with an average diameter size of 60 nm. From the TEM images and DLS, the average particle diameter of bare AgNPs and two silver Raman nanotags were found to be approximately 30, 50, and 45 nm, respectively.

To investigate the feasibility of simultaneous labelling of AFP and its fucosylation by the C-terminal epitope-imprinted nanotags and Fuc-imprinted nanotags, SEM characterization was carried out. AFP was first captured by an N-epitope-imprinted AuNP-coated substrate and then simultaneously labelled by C-epitope-imprinted nanotags and Fuc-imprinted nanotags, respectively. Nanotag pairs on the imprinted substrate were observed, and the number of nanotag pairs increased as the concentration of AFP tested was increased (Fig. S7A and B†). To exclude the possibility that the nanotag pairs were not formed from a single type of imprinted nanotags or the precipitation of two kinds of nanotags, control experiments were also performed. When either C-epitope-imprinted nanotags or Fuc-imprinted nanotags were used alone for the labelling, only single nanotags were observed on the substrate (Fig. S7C and D†). Moreover, when the sample test contained no AFP while two types of nanotags were applied for labelling, no nanotags were observed (Fig. S7E†). Lastly, if the two kinds of nanotags were applied for labelling but non-bound nanotags were not removed by washing, apparent precipitates of the nanotag were observed (Fig. S7F†). These results confirmed the feasibility of simultaneous labelling of AFP and its fucosylated glycoform with the two kinds of imprinted nanotags.

### Selectivity and affinity of anti-Fuc MIP and LCA lectin

AFP-L3 is one of the heterogeneous forms of AFP that could elevate diagnostic accuracy to 93% for HCC.^65,66^ The clinical method of AFP and AFP-L3 analysis relies on antibody and lectin-based immunoassay, using an anti-AFP antibody to capture AFP and its isoforms from real samples, then labelling the captured AFP isoforms with a secondary antibody for quantifying the captured AFP. For AFP-L3 quantification, the AFP isoforms captured by the antibody are labelled with a lectin, particularly LCA, which can recognize the core-fucosylated biantennary glycan on the heterogeneous of AFP. Since antibodies often have core-fucosylated glycans,^32^ the detection method of AFP-L3 based on ALSA for HCC diagnosis might have an uncontrolled blank value and inevitable interference. Besides, the inadequate specificity of lectin might result in poor measurement accuracy.

**Fig. 2:**
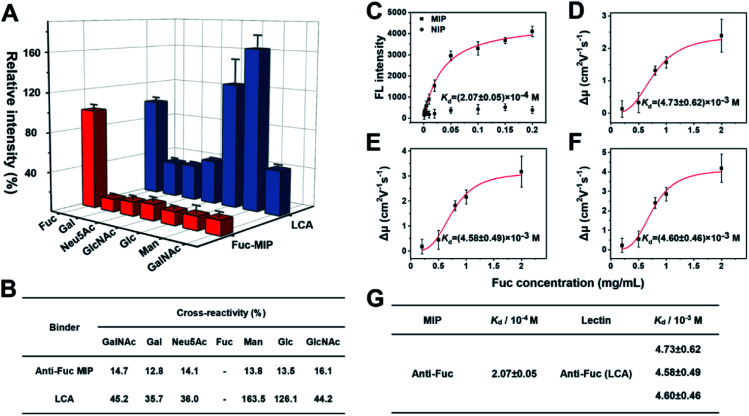
(A) Comparison of the selectivity of Fuc-MIP and LCA toward different monosaccharides. The error bars represent the standard deviation of three parallel experiments. (B) Comparison table of selectivity between MIP with lectin toward monosaccharides. (C) Adsorption isotherms of Fuc on Fuc-imprinted and non-imprinted NPs. (D)–(F) Fitting curves for the interactions between the three different isoforms of LCA with Fuc. Red curve, data fitting by the Hill equation. The error bars represent the standard deviation for three parallel experiments. (G) Comparison table of *K*_d_ values between Fuc-MIP and LCA lectin toward the target monosaccharide.

Fuc-imprinted MIP is key to the accuracy of triMIP-PISA toward HCC diagnosis. Therefore, the binding properties of Fuc-imprinted MIP were firstly in-depth investigated and compared with its competing agent, lectin LCA. For the convenience of easy operation, Fuc-specific MIP was prepared using MNPs as the substrate. Fuc-imprinted MNPs were prepared according to the boronate-affinity-controllable oriented surface imprinting approach.^64,67^ To immobilize the template onto the substrate for imprinting, functionalization of the core MNPs with boronic acid is essential. The boronate affinity of the core MNPs was investigated. As shown in Fig. S8,† the boronic acid-functionalized MNPs exhibited significant affinity toward *cis*-diol-containing compounds, including adenosine, ribonuclease B (RNase B) and horseradish peroxidase (HRP), as compared with non-*cis*-diol-containing, compounds, including deoxyladenosine, ribonuclease A (RNase A) and bovine serum albumin (BSA). This indicates that boronic acid was successfully modified onto the surface of MNPs. The cross-reactivities of the prepared anti-Fuc MIP towards different monosaccharides, including mannose (Man), galactose (Gal), *N*-acetylneuraminic acid (Neu5Ac), *N*-acetylglucosamine (GlcNAc), glucose (Glc) and *N*-acetylgalactosamine (GalNAc) were investigated and compared with those of LCA using the boronate affinity sandwich assay.^68^ For the test of LCA, boronic acid-immobilized 96-well plates were first prepared, and the test monosaccharides were then used as bridging reagents to bind with the boronic acid-functionalized 96-well plate, followed by labelling with the fluorescein-labelled LCA and fluorescence detection. As shown in Fig. 2A and B, LCA could bind not only Fuc but also Glc and Man even more (with cross-reactivity of 126.1% and 163.5%), which is consistent with the literature results.^69^ Also, its cross-reactivity towards other monosaccharides was high (at least 35.7%). In contrast, the anti-Fuc MIP exhibited significantly better selectivity toward the target monosaccharide, with cross-reactivity of less than 16.1% toward other monosaccharides.

Next, the affinity of anti-Fuc MIP and lectin toward different monosaccharides was investigated. To measure the *K*_d_ value of the anti-Fuc MIP, a series of Fuc standard solutions with known concentrations were incubated with an equivalent amount of anti-Fuc MIP. After the extraction, fucose molecules captured by the MIP were labelled with the fluorescence reagent 8-aminonaphthalene-1,3,6-trisulfonic acid disodium salt (ANTS), and the labelled captured Fuc molecules were released into an elution solution of appropriate volume. An isothermal adsorption curve was established by plotting the fluorescence intensity of Fuc molecules captured by the MIP against the concentration of Fuc in the standard solution. An isothermal adsorption curve for non-imprinted polymer material (NIP) was also constructed for comparison. As shown in Fig. 2C, as compared with the NIP, the MIP showed a typical isothermal curve for strong binding. The *K*_d_ value was calculated, by fitting the data according to the Hill equation, to be at the 10^−4^ M level. To measure the affinity between lectin and target monosaccharide, affinity capillary electrophoresis (ACE) was employed, and the *K*_d_ value was calculated by fitting the mobility changes of the lectin-monosaccharide complex against the corresponding concentration of monosaccharide added into the running buffer according to the Hill equation.^70^ In fact, LCA exhibited three isoforms in ACE, and their *K*_d_ values of lectin toward Fuc could be measured simultaneously (Fig. S9†). The Hill equation fitting for individual isoforms is shown in Fig. 2D–F, and a comparison of *K*_d_ values is shown in Fig. 2G. The *K*_d_ values of the three lectin isoforms towards Fuc were all at the 10^−3^ M level. Clearly, the anti-Fuc MIP exhibited better affinity towards Fuc. These results indicate that the anti-Fuc MIP was superior to the lectin LCA in terms of specificity and affinity toward the target monosaccharide. This superior binding property paves a robust basis for precise targeting of fucosylation. However, it should be noted that the recognition towards fucosylation on glycans may differ from free Fuc recognition, and the feasibility for real applications needs to be verified.

### Linear response curve for AFP and AFP-L3

**Fig. 3:**
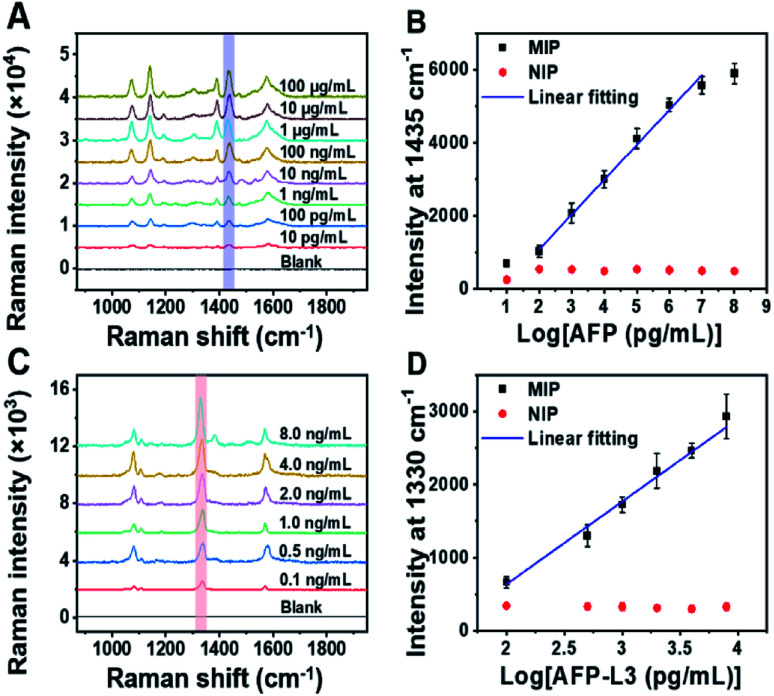
(A) Raman spectra of AFP standard at different concentrations detected by MIP-PISA. (B) Concentration-dependent Raman intensity of AFP N-terminal and C-terminal epitopes detected by MIP-PISA approach and the corresponding NIP-PISA on the logarithm of AFP concentration, AFP linearity range: 0.1 ng mL^−1^–10 μg mL^−1^, *y* = −816.9 + 952.3*x*, *R*^2^ = 0.99. (C) Raman spectra of AFP-L3 standard at different concentrations detected by the MIP-PISA. (D) Concentration-dependent Raman intensity of AFP N-terminal epitope and Fuc detected by MIP-PISA approach and corresponding NIP-PISA on the logarithm of the AFP-L3 concentration, AFP-L3 linearity range: 0.1–8 ng mL^−1^, *y* = −1626.9 + 1131.7*x*, *R*^2^ = 0.99. The error bars represent the standard deviation for three parallel experiments.

To quantify total AFP and AFP-L3 in real samples, calibration curves for AFP and AFP-L3 were established. As shown in Fig. 3, two calibration curves were obtained by plotting the signal intensity of the Raman characteristic peaks against the logarithm of the concentration of total AFP and AFP-L3, which obeyed good linear relationships. For the quantitation of total AFP, the linear relationship was *y* = −816.9 + 952.3*x*, *R*^2^ = 0.99, while for the quantitation of AFP-L3, the linear relationship was −1626.9 + 1131.7*x*, *R*^2^ = 0.99.

### Real sample analysis for precise diagnosis of HCC *via* triMIP-PISA

**Fig. 4:**
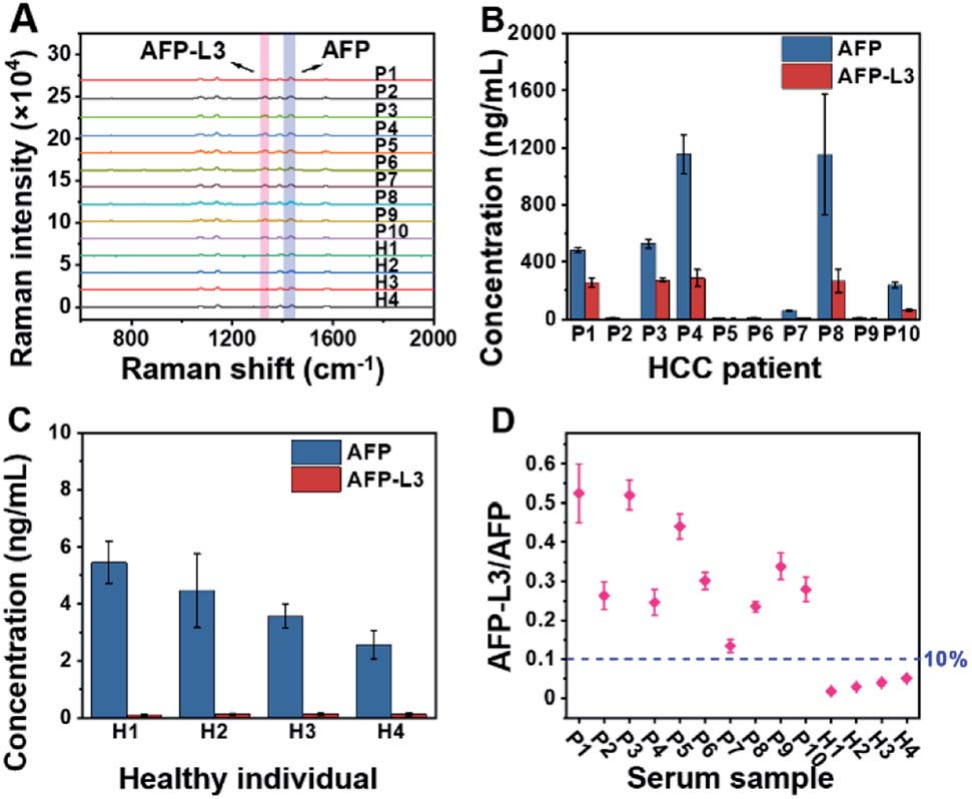
(A) Raman spectra for AFP and AFP-L3 in the serum samples of HCC patients and healthy individuals (several Raman spectra are the response of diluted samples from patients; see the ESI† section for specific dilutions). (B) Concentration histogram of HCC patients' serum. (C) Concentration histogram of healthy individuals' serum. The error bars represent the standard deviation for three parallel experiments (P: patient, H: healthy). (D) Scatter plot of the AFP-L3/AFP ratio for serum samples of HCC patients and healthy individuals.

To demonstrate the feasibility of the established triMIP-PISA method for real sample applications, 14 clinical serum samples, including 10 from HCC patients and 4 from healthy individuals, were analyzed. Representative Raman spectra for these samples are shown in Fig. 4A. The concentrations of total AFP and AFP-L3 in these serum samples were determined according to the calibration curves, and the results are listed in Table S1.†Fig. 4B and C show the concentrations of total AFP and AFP-L3 of HCC patients and healthy individuals, respectively. It can be seen that the concentrations of total AFP and AFP-L3 varied from sample to sample. Also, it is clear that there was no obvious cut-off value for the concentration of AFP between the HCC patients and the healthy individuals. This is in agreement with the verified conclusion that the diagnosis of liver cancer in terms of total AFP alone is not accurate.^71,72^ In fact, 10% of the AFP-L3/AFP ratio has been suggested as the criteria for precise diagnosis of HCC from chronic liver disease in clinical practice.^73^ Thus, the ratios of AFP-L3/AFP for HCC patients and healthy individuals are listed together and compared. As shown in Fig. 4D, there is a clear boundary between HCC patients' samples and healthy individuals' samples, located at about 10% of the ratios. This is consistent with the clinical cut-off value used to distinguish HCC patients from healthy individuals. This suggests that our triMIP-PISA method can be a reliable alternative to the current clinical methods for precise diagnosis of HCC.

### Comparing the precision of triMIP-PISA with LCA-IFA

**Fig. 5:**
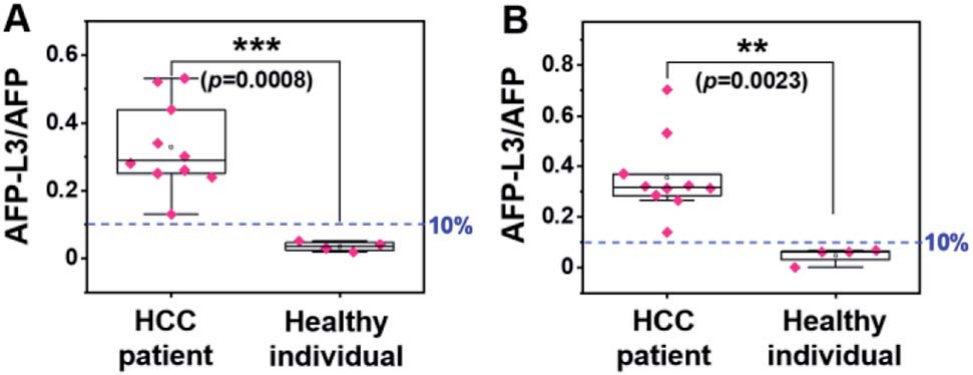
Box plot for differentiating HCC patients from healthy individuals according to the results by (A) triMIP-PISA and (B) LCA-IFA.

Using the same set of samples, we further compared the precision of our method with an LCA-immunofluorescence assay (LCA-IFA), which has been approved for clinical detection of AFP-L3 over total AFP. To avoid the influence of operator on the analysis, LCA-IFA of the 14 samples was carried out at an independent clinical laboratory. The results measured by LCA-IFA are also listed in Table S1† and compared with those from our triMIP-PISA. As shown in Table S1,† the results from LCA-IFA are different from those of our triMIP-PISA, and the difference varied from sample to sample. The dataset was further statistically analyzed using a box plot. As shown in Fig. 5, according to the ratio of AFP-L3/AFP, both methods allowed for differentiation of HCC patients from healthy individuals. In terms of the statistical significance, our triMIP-PISA showed higher accuracy over LCA-IFA, with a *p*-value of 0.0008 as compared with 0.023 for the latter. The much lower probability value suggests that our method is a more precise tool for early diagnosis of HCC. Besides, compared to the LCA-IFA approach, the method reported in this study exhibited several significant advantages, including a lower sample volume requirement (5 μL *vs.* 100 μL), a simpler procedure (5 steps *vs.* 7 steps) and a wider linear range for AFP (0.1 ng mL^−1^ to 10 μg mL^−1^*vs.* 0.3–2000 ng mL^−1^).

## Conclusions

In summary, we have developed a triMIP-based PISA strategy for precise early disease diagnosis according to the relative glycosylation level of glycoprotein biomarkers. In this approach, an epitope-imprinted MIP enabled the specific extraction of target glycoprotein from clinical samples, and another epitope-imprinted MIP allowed the specific labelling and further recognition of the captured target molecules; meanwhile, a monosaccharide-imprinted MIP specifically reported the presence of specific glycans of the target glycoprotein. Moreover, PERS detection permitted the simultaneous and ultrasensitive measurement of the peptide level and the glycan level. Through simultaneously measuring the AFP-L3 and AFP levels in human serum, more precise diagnosis of HCC patients from healthy individuals over the clinically approved LCA-immunofluorescence assay was achieved. The improved diagnosis precision benefited greatly from the excellent recognition properties of the MIPs employed, particularly the binding properties of the monosaccharide-imprinted MIP, which are superior to those of lectins. In fact, MIPs specific to other monosaccharides have already been prepared using the same imprinting approach and verified to outperform their lectin counterparts (data not shown). Since many disease biomarkers are glycoproteins and monosaccharide-targeting MIPs enabled specific detection of their aberrant glycans, the strategy presented herein can be extended to many other disease biomarkers. Thus, the presented strategy provides a new avenue for precise early diagnosis of diseases.

Since this triMIP-based PISA strategy is new, a few basic aspects remain to be investigated. In particular, for more practical applications, the size of the labelling nanotags should be reduced to an appropriate level to avoid or reduce steric hindrance that may prevent the nanotags from labelling the target. This is critical for a glycoprotein biomarker with a small size or too-close epitope and glycan to be labelled. When steric hindrance is inevitable, a possible solution is to perform two parallel assays on two spots and to label and detect the epitope and glycan separately, whereas another one may be to stretch the captured target glycoprotein into a linear peptide chain by denaturing.

## Data availability

Most of experimental data have been shown in the manuscript. Original data are available upon request from the corresponding author with appropriate reason.

## Author contributions

Z. Liu conceived the idea and supervised, wrote and reviewed the manuscript. J. Pang carried out the experiments and drafted the manuscript. P. Li performed the material optimization and discussion. H. He assisted in glycan analysis and discussion. S. Xu helped with the sample collection and discussion.

## Conflicts of interest

There are no conflicts to declare.

## Supplementary Material

SC-013-D2SC01093C-s001
