# Supplementary material for: Molecularly imprinted polymers outperform lectin counterparts and enable more precise cancer diagnosis
Source: Chem Sci. 2022 Mar 17;13(16):4589–97. doi: 10.1039/d2sc01093c (PMC9020343; doi:10.1039/d2sc01093c)
Supplement: SC-013-D2SC01093C-s001 [file SC-013-D2SC01093C-s001.pdf]

Electronic Supplementary Material (ESI) for Chemical Science.
This journal is © The Royal Society of Chemistry 2022

Electronic Supplementary Material (ESI) for Chemical Science.
This journal is © The Royal Society of Chemistry 2022

Electronic Supplementary Information for

Molecularly Imprinted Polymers Outperform Lectin Counterparts and Enable More Precise Cancer Diagnosis

Jilei Pang[#]*, Pengfei Li*[#]*, Hui He, Shuxin Xu, Zhen Liu**

State Key Laboratory of Analytical Chemistry for Life Science, School of Chemistry and Chemical Engineering, Nanjing University, Nanjing 210023, China

[*] Corresponding author, Email: zhenliu@nju.edu.cn.

[#] Equally contributed authors.

Contents:

Materials and methods

Reagents and Materials

Apparatus

Methods

Supplementary Data

Fig. S1 to Fig. S9

Table S1

Materials and methods

1. Reagents and Materials

Alpha-fetoprotein (AFP) was purchased from Shanghai Dakewei Biotechnology (Shanghai, China). AFP-L3 was purchased from Jiangsu Meimian Industrial Technology (Yancheng, China). Horseradish peroxidase (HRP), bovine serum albumin (BSA), ribonuclease A (RNase A), ribonuclease B (RNase B), dimethyl sulfoxide (DMSO, 99%), 2,4-difluoro-3-formylphenyl-boronic acid (DFFPBA, 99%), adenosine (A, 99.5%), deoxyadenosine (DA, 99.5%), 4-aminothiophenol (PATP, 97%) and 4-nitrothiophenol (NTP, 80%) were obtained from Sigma-Aldrich (St. Louis, MO, USA). Methanol and acetonitrile (ACN) and N-Acetylneuraminic acid (Neu5Ac, 98%) were purchased from Macklin Biochemical (Shanghai, China). L-fucose (Fuc, 99%), N-Acetyl-D-galactosamine (GalNAc, 98%) was purchased from Aladdin Industrial (Shanghai, China). D-Glucose (Glc, 99%), D-Mannose (Man, 99%), D-Galactose anhydrous (Gal, 99%), N-Acetyl-D-glucosamine (GlcNAc, 98%), 4-Formylphenylboronic acid (FPBA, 97%), sodium cyanoborohydride ($NaBH_3CN$, 95%) 3-aminopropyltriethoxysilane (APTES, 98%), 3-ureidopropyltriethoxysilane (UPTES, 50%), isobutyltriethoxysilane (IBTES, 98%) and 8-aminonaphthalene-1,3,6-trisulfonic acid disodium salt (ANTS, 99%) were purchased from J&K Chemical (Shanghai, China). Benzyltriethoxysilane (BnTES) and ammonia solution (28%) were purchased from Sinopharm Chemical Reagent (Shanghai, China). Fructosylated peptides with HPLC purity of 98%, including Fru-RTLHRNEYGIAS and KLISKTRAALGVK-Fru, were custom-ordered from Top-Peptide Biotechnology (Shanghai, China). Fluorescein-labeled Lens Culinaris Agglutinin (LCA) was purchased from Vector Laboratories (Burlingame, USA). Tetraethylorthosilicate (TEOS, 99%) was purchased from Heowns Biochemical Technology (Tianjin, China). Ferric trichloride hexahydrate, 1,6-hexanediamine, anhydrous sodium acetate, glycol, silver nitrate ($AgNO_3$), ammonium bicarbonate, sodium dihydrogen phosphate, sodium hydroxide, sodium

chloride (NaCl), acetic acid (HAc) and anhydrous ethanol were purchased from Nanjing Reagent Company (Nanjing, China). Trisodium citrate was purchased from Shanghai Lingfeng Chemical Reagent (Shanghai, China). All other chemical reagents were of analytical grade unless otherwise stated. Water used in all experiments was purified by a Milli-Q Advantage A10 ultrapure water purification system (Millipore, Milford, MA, USA). Serum samples from HCC patients and healthy individuals were obtained from Jiangsu Province Hospital (Nanjing, China) and approved by the institutional ethics committee of Jiangsu Province Hospital. All serum samples were isolated, aliquoted, and stored at -20°C until use.

2. Apparatus

Transmission electron microscopic (TEM) and scanning electron microscope (SEM) characterization were carried out on a JEM-2100 system (JEOL, Tokyo, Japan) and S-4800 (Hitachi, Japan). Ultraviolent (UV)-vis spectral analysis was performed with a NanoDrop One spectrophotometer (ThermoFisher, MA, USA). Characterization of particle sizes was carried out on a BI 200SM dynamic light scattering (DLS) instrument (Brookhaven Instrument, Holtsville, USA). Fluorescence spectral analysis was performed on a Synergy Mx microplate reader from BioTek (Winooski, VT, USA). Capillary electrophoresis (CE) experiments were conducted on a P/ACE MDQ system (Beckman Coulter, Fullerton, CA, USA) equipped with a diode array detector (DAD) and a UV detector. A fused-silica capillary of 75 μm i.d. × 60 cm (50 cm to the detector) from Yongnian Optical Fiber Factory (Hebei, PRC) was used as the separation column. Unless specified, CE separations were performed at 25°C under optimum voltage settings (typically 20 KV) and UV absorbance was acquired using at the wavelength of 214 nm. Prior to each run, the capillary was sequentially rinsed at 20 psi with 0.1 M NaOH for 3 min and running buffer for 3 min. Samples were injected by pressure at 0.5 psi for 5 s. Raman detection was carried out on an XploRA Plus confocal Raman microscope controlled by the software LabSpec 6 (Horiba, France). Spectra were acquired using a 638 nm excitation laser

line (energy level about 10%). The laser beam was focused onto samples deposited on the arrays via a ×40_FLUOR objective lens. Wavelength calibration was performed by measuring silicon wafers through a × 40_FLUOR objective before the test. Each spectrum was baseline corrected except for the noise test.

3. Methods

3.1 Preparation of gold nanoparticles (AuNPs)

AuNPs were synthesized as a previously described method.[1] Briefly, 0.5 mL of $HAuCl_4$ (1%, w/v) was added in 50 mL of water, and brought to a boil with vigorous stirring, and then 0.424 mL of sodium citrate solution (34 mM) was rapidly added to the above solution. After the color of the solution changed from colorless to purple, the solution was continued to reflux for 15 min, then cooled down to room temperature under stirring conditions. Finally, the obtained Au colloidal solution was stored at 4°C before use.

3.2 Boronic acid-functionalization of AuNPs self-assembled monolayer-coated substrates

To prepare epitope-imprinted plasmonic arrays for specifically capturing and enriching AFP from the samples, boronic acid-functionalized AuNPs self-assembled monolayer (SAM)-coated glass substrates were needed to prepare in advance. The preparation route was comprised of three steps: 1) amino-functionalization of glass substrates, 2) amino-functionalization of AuNPs SAM coated-glass substrates, and 3) boronic acid-functionalization of AuNPs SAM coated-glass substrates.

For the preparation of amino-functionalized glass substrates, microscope glass slides (75 mm × 25 mm) were firstly cut into equal pieces (25 mm × 10 mm) and then four small circles with a diameter of 4 mm were carved on the surface of each piece. The obtained uniformly sized substrates were immersed in piranha solution (H_2SO_4: H_2O_2 = 7: 3, v/v) for 1 h, and then rinsed repeatedly with water and anhydrous ethanol, and dried under a nitrogen stream. To

functionalize with amino groups, the obtained substrates were soaked in 4% (v/v) APTES solution dissolved in anhydrous ethanol for 9-12 h at room temperature. The resulting amino-functionalized glass substrates were rinsed repeatedly with anhydrous ethanol and water to remove the unreacted APTES from the surface of the glass substrates.

To prepare amino-functionalized AuNPs SAM coated-glass substrates, the amino-modified glass substrates were soaked in 5 mL of the Au colloidal solution for 12 h at room temperature, and then washed with water to remove the unbound AuNPs. The amino group of APTES could bind with AuNPs by electrostatic interaction, so a monolayer of AuNPs was formed on the surface of the glass substrates. The obtained AuNPs SAM coated-glass substrates were soaked in 4% (v/v) APTES solution dissolved in anhydrous ethanol for 2 h at room temperature. The resulting amino-functionalized AuNPs SAM coated-glass substrates were rinsed repeatedly with anhydrous ethanol and water to remove the unreacted APTES.

To prepare boronic acid-functionalized AuNPs SAM coated-glass substrates, the amino-functionalized AuNPs SAM coated-glass substrates were soaked in 5 mL of methanol containing 5.0 mg/mL FPBA and 1.0 mg/mL $NaBH_3CN$. After reaction at room temperature for 24 h, the obtained FPBA-functionalized AuNPs SAM coated-glass slide was washed repeatedly with anhydrous ethanol and water. The prepared boronic acid-functionalized AuNPs SAM-coated glass substrates were stored at 4°C for later use.

3.3 Preparation of AFP N-terminal epitope-imprinted arrays

To prepare AFP N-terminal epitope-imprinted plasmonic arrays for specifically capturing and enriching AFP from the samples, boronic acid-functionalized AuNPs SAM-coated glass substrates were needed to prepare in advance.

The imprinting approach for the preparation of AFP N-terminal epitope-imprinted arrays was the same as our previous method,[2] except that the nanoparticle substrate was replaced with

SAM-coated glass substrate. The procedure is illustrated in Fig. S3. The detailed processes are described as follows.

For the immobilization template of AFP N-terminal epitope, 5 μL of 1.0 mg/mL glycated AFP N-terminal epitope solution dissolved in 50 mM ammonium bicarbonate buffer (pH 8.5) containing 150 mM NaCl was first added onto each spot of the boronic acid-functionalized AuNPs SAM-coated arrays. Then the arrays were incubated for 2 h in a humidity chamber to form a thin template layer. During this period, ammonium bicarbonate buffer (50 mM, pH 8.5) containing 150 mM NaCl was supplemented every 20 min. The obtained glycated AFP N-terminal epitope-immobilized AuNPs SAM-coated arrays were rinsed with 50 mM ammonium bicarbonate buffer (pH 8.5) containing 150 mM NaCl three times.

For oriented surface imprinting, the template-anchored arrays were immersed in a mixture of 3 mL of anhydrous ethanol containing 90 μL of ammonium hydroxide (28%) and 0.2 mL of water. Then monomers (APTES: UPTES: BnTES: IBTES: TEOS=20: 20: 10: 20: 30,) of the desired molar ratio were dissolved in 0.8 mL of anhydrous ethanol, and then the resulting solution was added to the above suspension and mixed evenly. Further, the obtained epitope-bound AuNPs SAM-coated arrays were quickly immersed in the mixed solution and reacted for 50 min at 25°C. Then, the obtained arrays were washed with absolute ethanol and water three times each, to remove unpolymerized monomers. For the cladding processing, the arrays were immersed in the mixed solution of 4 mL of anhydrous ethanol containing 56 μL of ammonium hydroxide (28%) and 0.8 mL prepolymer solution (consisted of 22.4 μL TEOS and 10 mL anhydrous ethanol) to react for 10 min at 25°C. After that, the obtained arrays were washed with anhydrous ethanol and water three times each.

To remove the template, the arrays were rinsed with 4 mL of a mixed solution of acetonitrile, water and acetic acid (50: 49: 1, v/v) for 3h at room temperature, followed by washing with 4 mL of anhydrous ethanol and water for three times each. Non-imprinted arrays

were prepared using the same procedure except for the absence of the template immobilization step. The prepared imprinted and non-imprinted arrays were stored at 4°C before use.

3.4 Preparation of silver nanoparticles (AgNPs)

A 700-μL volume of 10 mM ascorbic acid was dissolved in 47 mL of water in a 250-mL three-neck round-bottom flask. The solution boiled under continuous stirring. Then, 2 mL water, 1.4 mL of 1% (w/v) trisodium citrate and 600 μL of 1% $AgNO_3$ were added to the above flask. The mixture was boiled with stirring for about 60 min and then cooled down to room temperature naturally. The obtained AgNPs colloidal solution was stored at 4°C before use.

3.5 FPBA-functionalization of Ag/PATP (or NTP) @SiO_2 NPs

FPBA-functionalized Ag/PATP@SiO_2 NPs were prepared according to our previously reported method[3] with slight modifications. The preparation procedure was comprised of three steps: 1) synthesis of Ag/PATP (or NTP) @SiO_2 NPs, 2) amino-functionalization of Ag/PATP (or NTP) @SiO_2 NPs, and 3) boronic acid-functionalization of Ag/PATP (or NTP) @SiO_2 NPs with FPBA.

For the synthesis of Ag/PATP (or NTP) @SiO_2 NPs, 40 μL of 1 mM PATP (or NTP) dissolved in anhydrous ethanol was first added dropwise to 20 mL of Ag colloidal solution under rapid stirring for 40 min. Then 40 mL of anhydrous ethanol and 1.2 mL of ammonia solution (28%) were added to the above solution, respectively, and the mixture was stirred for 5 min. A 20-mL volume of prepolymer solution consisting of 20 μL TEOS and 20 mL anhydrous ethanol was added to the above suspension, and the mixture was stirred at room temperature for 60 min. After that, the resultant Ag/PATP (or NTP) @SiO_2 NPs were centrifuged at 10,000 rpm for 10 min and washed with anhydrous ethanol three times. The Ag/PATP (or NTP) @SiO_2 NPs were redispersed in 20 mL of anhydrous ethanol.

For the preparation of amino-modified Ag/PATP (or NTP) @SiO_2 NPs, 200 μL of APTES was added to 20 mL of Ag/PATP (or NTP) @SiO_2 NPs suspension dissolved in anhydrous ethanol, and the mixture was stirred at room temperature for 1 h. The resulting amino-modified Ag/PATP (or NTP) @SiO_2 NPs were centrifuged at 10,000 rpm for 10 min, and then washed with anhydrous ethanol three times. The amino-modified Ag/PATP (or NTP) @SiO_2 NPs were redispersed in 30 mL of methanol.

To prepare FPBA-functionalized Ag/PATP (or NTP) @SiO_2 NPs, 300 μL of 5.0 mg/mL FPBA and 300 μL of 1.0 mg/mL $NaBH_3CN$ were added into 30 mL of amino-modified Ag/PATP (or NTP) @SiO_2 NPs suspension. After reaction for 24 h, the obtained FPBA-functionalized Ag/PATP (or NTP) @SiO_2 NPs were centrifuged at 10,000 rpm for 10 min, and then washed with anhydrous ethanol and water three times each. The FPBA-functionalized Ag/PATP (or NTP) @SiO_2 NPs were redispersed in 9 mL of 10 mM phosphate buffer (pH 7.4).

3.6 Preparation of AFP C-terminal epitope-imprinted Ag/PATP@SiO_2 NPs

To prepare AFP C-terminal epitope-imprinted plasmonic tags for specifically labeling AFP captured by AFP N-terminal epitope-imprinted arrays from the samples, boronic acid-functionalized Ag/PATP@SiO_2 NPs were needed to prepare in advance. The imprinting approach for the preparation of AFP C-terminal epitope-imprinted Ag/PATP@SiO_2 NPs was the same as our previous method,[2] except that the template was changed to glycated AFP C-terminal epitope template while the monomer ratio and imprinting time were re-optimized using similar procedure. The procedure is illustrated in Fig. S4. The detailed processes are described as follows.

To immobilize the templates, 9 mL FPBA-functionalized Ag/PATP@SiO_2 NPs were dispersed by ultra-sonication. Then 1 mL of 1 mg/mL AFP C-terminal glycated epitope solution (solvent: pH 7.4 10 mM phosphate buffer) was added to the suspension and shaken at room temperature for 2 h. The obtained AFP C-terminal glycated epitope-immobilized

$Ag/PATP@SiO_2$ NPs were collected via centrifuging and washed with 10 mM phosphate buffer (pH 7.4).

To imprint AFP C-terminal epitope on the AgNPs, the collected AFP C-terminal glycated epitope bound $Ag/PATP@SiO_2$ NPs were dispersed into 16 mL anhydrous ethanol containing 0.45 mL ammonium hydroxide (28%) and 1 mL water. Then monomers (APTES: UPTES: IBTES: TEOS=10: 20: 40: 30,) of the desired molar ratio were dissolved in 4 mL of anhydrous ethanol, and then the resulting solution was added to the above suspension and the mixture was gently stirred at 25°C for 60 min. After that, the obtained AFP C-terminal epitope-imprinted $Ag/PATP@SiO_2$ NPs were washed with anhydrous ethanol and water three times each. For the cladding processing, the particles were dispersed in the mixed solution of 20 mL of anhydrous ethanol containing 280 μL of ammonium hydroxide (28%) and 4 mL prepolymer solution (consisted of 22.4 μL TEOS and 10 mL anhydrous ethanol) then reacted for 10 min at 25°C. Finally, the obtained nanoparticles were washed with anhydrous ethanol and water three times each.

To remove the epitope template, the obtained AFP C-terminal epitope-imprinted $Ag/PATP@SiO_2$ NPs were dispersed into 10 mL of ACN: H_2O: HAc = 50: 49: 1 (v/v) and shaken for 60 min at room temperature. The above elution process was repeated three times. After removing the epitope templates, the prepared epitope-imprinted MNPs were collected by centrifugation, washed with water and anhydrous ethanol three times each and then dispersed in 0.2 mL 10 mM phosphate buffer (pH 7.4) for further use. Non-imprinted particles were prepared using the same procedure except for the absence of glycated epitope template.

3.7 Optimization of monomer ratio and imprinting time for the imprinting of C-terminal epitope of AFP

To obtain high-performance imprinted materials, the monomer ratio and imprinting time for the preparation of C-terminal epitope-imprinted MIP were optimized. The monomer ratio

and imprinting time were optimized according to the obtained imprinting factor (IF). A series of AFP C-terminal epitope-imprinted and non-imprinted MNPs were prepared according to the procedure described our previous method,[2] except that proportions of monomers (APTES, UPTES, IBTES and TEOS) and imprinting time were specified as in Fig. 1A. 2.0 mg of the AFP C-terminal epitope-imprinted and non-imprinted MNPs prepared by different proportions of monomers (APTES, UPTES, IBTES and TEOS) at different imprinting time were added to 1 mL of 0.1 mg/mL epitope dissolved in 10 mM phosphate buffer (pH 7.4), respectively. After incubation at room temperature for 30 min, the MNPs were magnetically collected and rinsed with 1 mL of 10 mM phosphate buffer (pH 7.4) three times. The MNPs were re-suspended and eluted in 50 μL of ACN: H_2O: HAc = 50: 49: 1 (v/v) for 60 min on a rotator. Finally, the MNPs were magnetically separated and the eluates were collected. The amount of epitope in the eluates was determined via measuring the UV absorbance at 214 nm. The detection was repeated three times. The obtained optimization results for the preparation of high-performance imprinted materials were used as C-terminal epitope Raman nanotags for labeling captured AFP.

3.8 Selectivity test of AFP C-epitope-imprinted MIP

The selectivity of the AFP C-terminal epitope-imprinted MIP toward different proteins including AFP, HRP, BSA, TRF and β-casein was evaluated using the AFP C-terminal epitope-imprinted MNPs to replace the same template-imprinted SERS nanotags due to easy magnetic separation. First, a 0.1 mg/mL solution of each protein was separately prepared with 10 mM phosphate buffer (pH 7.4). Then equivalent epitope-imprinted MNPs and non-imprinted MNPs (2 mg each) were added to 1 mL of the protein solutions in 1.5-mL centrifugal tubes, respectively. The tubes were shaken on a rotator at room temperature for 30 min. The MNPs were magnetically collected and rinsed with 1 mL of 10 mM phosphate buffer (pH 7.4) three times. Second, the MNPs were re-suspended and eluted in 50 μL of ACN: H_2O: HAc = 50: 49: 1 (v/v) for 60 min on a rotator. Finally, the MNPs were magnetically separated and the eluates

were collected. The amount of captured proteins on the epitope-imprinted MNPs were determined through measuring the UV absorbance of the eluates at 214 nm. The detection was repeated three times.

3.9 Preparation of Fuc-imprinted Ag/NTP@SiO_2 NPs

To prepare Fuc-imprinted Ag/NTP@SiO_2 NPs for specifically labeling the fucosylated glycans of AFP captured by AFP N-terminal epitope-imprinted arrays from the samples, boronic acid-functionalized Ag/NTP@SiO_2 NPs were needed to prepare in advance.

The Fuc-imprinted Ag/NTP@SiO_2 NPs was prepared according to our previously developed method.[4,5] The procedure is illustrated in Fig. S5. To immobilize the template Fuc onto boronic acid-functionalized Ag/NTP@SiO_2 NPs, 1.0 mL of 1 mg/mL Fuc solution dissolved in 0.01 M phosphate buffer (pH 7.4) was added into 9 mL the prepared boronic acid-functionalized Ag/NTP@SiO_2 NPs and the pH was adjusted to 7.4. After incubation for 2 h, Fuc-bound SiO_2 NPs were collected via centrifuging and then washed with 0.01 M phosphate buffer (pH 7.4) three times. The Fuc-bound Ag/NTP@SiO_2 NPs were re-dispersed into 5.0 mL ethanol, added with 90 μL ammonium water and 1.25 mL prepolymer solution that was consisted of 22.4 μL TEOS and 10 mL anhydrous ethanol. After imprinting for 15 min at 25°C, the reacting mixture was centrifuged and the precipitates were collected. Finally, to remove the template from the imprinted nanoparticles, the collected precipitates were washed with 0.1 M HAc for 3 h, followed with 0.01 M phosphate buffer for 30 min and centrifugation again. The obtained Fuc-imprinted Ag/NTP@SiO_2 NPs were collected and stored in 0.01 M phosphate buffer (pH 7.4).

3.10 Preparation of magnetic nanoparticles (MNPs)

Fe_3O_4 MNPs were synthesized according to a previously reported method.[3] Briefly, 2.0 g of ferric trichloride hexahydrate, 13.0 g of 1,6-hexanediamine and 4.0 g of anhydrous sodium

acetate were mixed with 60 mL glycol in a PTFE-lined autoclave and reacted at 198°C for 6 h. The resulting Fe_3O_4 MNPs were washed with water and anhydrous ethanol for three times each, and then dried at 50°C in a vacuum oven overnight.

3.11 Preparation of boronic acid-functionalized $Fe_3O_4@SiO_2$ MNPs

DFFPBA-functionalized $Fe_3O_4@SiO_2$ MNPs were synthesized via a process comprised of three steps: 1) synthesis of $Fe_3O_4@SiO_2$ MNPs, 2) amino-functionalization of $Fe_3O_4@SiO_2$ MNPs, 3) boronic acid-functionalization of $Fe_3O_4@SiO_2$ MNPs. The detailed procedures are described below.

3.11.1 Synthesis of $Fe_3O_4@SiO_2$ MNPs

200 mL of anhydrous ethanol, 7.5 mL of ammonium hydroxide (28%) and 1.4 mL of TEOS were added into a 500-mL three-neck round-bottomed flask, and then mechanically stirred at 400 rpm for 20 min at 40°C. 200 mg of MNPs were dispersed into 20 mL of anhydrous ethanol by ultra-sonication. The obtained suspension was added into the above flask, and then mechanically stirred at 400 rpm for 20 min at 40°C. The resulting $Fe_3O_4@SiO_2$ MNPs were collected by a magnet, washed with water and anhydrous ethanol three times each, and then dried at 50°C in a vacuum oven overnight.

3.11.2 Amino-functionalization $Fe_3O_4@SiO_2$ MNPs

The obtained $Fe_3O_4@SiO_2$ MNPs were dispersed into 100 mL of anhydrous ethanol by ultra-sonication in a 250-mL three-neck round-bottomed flask. Then 3 mL of APTES was added into the flask, and mechanically stirred at 400 rpm for 12 h at 80°C in a water bath. The resulting amino-functionalized $Fe_3O_4@SiO_2$ MNPs were collected by a magnet, washed with water and anhydrous ethanol three times each, and then dried at 50°C in a vacuum oven overnight.

3.11.3 DFFPBA-functionalization of $Fe_3O_4@SiO_2$ MNPs

A 200-mg amount of amino-functionalized Fe_3O_4@SiO_2 MNPs was added to 80 mL of methanol containing 400 mg of DFFPBA and 1% (w/w) $NaBH_3CN$ in a 250-mL three-neck round-bottomed flask, then the mixture was mechanically stirred at 400 rpm for 24 h at room temperature. The obtained Fe_3O_4@SiO_2@DFFPBA MNPs were collected by a magnet, washed with water and anhydrous ethanol three times each, and then dried at 50°C in a vacuum oven overnight. The obtained Fe_3O_4@SiO_2@DFFPBA MNPs were stored in a dry and sealed tube at room temperature for further use.

3.12 Selectivity test of boronic acid-functionalized Fe_3O_4@SiO_2 MNPs

The selectivity of Fe_3O_4@SiO_2@DFFPBA MNPs was investigated using adenosine and deoxyadenosine as test compounds. 2 mg of Fe_3O_4@SiO_2@DFFPBA MNPs was dispersed into 200 μL of 1.0 mg/mL adenosine or deoxyadenosine in ammonium bicarbonate buffer (50 mM, pH 8.5) containing 150 mM NaCl, then the mixture was shocked on a rotator at room temperature for 2 h. The Fe_3O_4@SiO_2@DFFPBA MNPs were magnetically collected and rinsed with 200 μL of ammonium bicarbonate buffer (50 mM, pH 8.5) containing 150 mM NaCl. Finally, the Fe_3O_4@SiO_2@DFFPBA MNPs were re-suspended and eluted in 20 μL of 0.1 M HAc solution for 1 h on a rotator. The Fe_3O_4@SiO_2@DFFPBA MNPs were magnetically separated and the eluates were collected. The amounts of adenosine or deoxyadenosine bound by the Fe_3O_4@SiO_2@DFFPBA MNPs were determined by measuring the amounts of adenosine or deoxyadenosine in the eluates according to UV absorbance at 260 nm. The measurement was repeated three times. Subsequently, two glycoproteins (RNase B and HRP) and two non-glycoproteins (RNase A and BSA) were selected as test protein samples. The procedure was the same as above except that the eluates were measured at 214 nm for UV absorbance.

3.13 Preparation of monosaccharide-imprinted MNPs

The preparation of monosaccharide-imprinted MNPs included three steps: 1) template immobilization, 2) oriented imprinting, and 3) template removal. The detailed procedures are described below.

3.13.1 Immobilization of monosaccharide template

Fuc was used as the monosaccharide template to be immobilized onto boronic acid-functionalized MNPs. 2 mg of Fuc template was dissolved in 2 mL of phosphate buffer (0.1 M, pH 7.4). Then 20 mg of Fe_3O_4@SiO_2@DFFPBA MNPs was dispersed in the resulting solution by ultra-sonication, and then stirred at room temperature for 2 h. The obtained Fuc-bound Fe_3O_4@SiO_2@DFFPBA MNPs was magnetically collected and then washed with phosphate buffer (0.1 M, pH 7.4) three times.

3.13.2 Oriented imprinting

Fuc-imprinted Fe_3O_4@SiO_2@DFFPBA MNPs was prepared according to our previous approach called boronate affinity oriented surface imprinting.[4-6] Briefly, Fuc-bound MNPs was re-dispersed into 160 mL ethanol, added with 2.8 mL ammonium water and 40 mL prepolymer solution that was consisted of 89.6 μL TEOS and 40 mL anhydrous ethanol. The mixture was mechanically stirred at 25°C for 15 min, which was the imprinting time for Fuc template. The obtained Fuc-imprinted Fe_3O_4@SiO_2@DFFPBA MNPs was collected by a magnet, washed with anhydrous ethanol three times, and then dried at 40°C in a vacuum oven overnight.

3.13.3 Removal of monosaccharide template

The obtained Fuc-imprinted Fe_3O_4@SiO_2@DFFPBA MNPs was dispersed into 2 mL of 0.1 M HAc and shaken for 3 h at room temperature. After removing the template molecules, the prepared Fuc-imprinted MNPs was magnetically collected, washed with water and anhydrous ethanol three times each and then dried at 40°C in a vacuum oven overnight. Non-

imprinted (NIP) MNPs were prepared using the same procedure except for the absence of monosaccharide templates.

3.14 Selectivity test of Fuc-imprinted MNPs

Using Fuc-imprinted MNPs to replace the same template-imprinted SERS nanotags for easy magnetic separation. Preparation of magnetic nanoparticles (MNPs), boronic acid-functionalized $Fe_3O_4@SiO_2$ MNPs and imprinting procedures are described above.

The selectivity of Fuc-imprinted and non-imprinted MNPs were investigated and Neu5Ac, Glc, Man, Gal, GalNAc, and GlcNAc were used as competing monosaccharides. A 2-mg amount of Fuc-MIP MNPs was added into a 0.5-mL centrifuge tube, each tube was added with a 100-μL solution of different monosaccharides (50 mM phosphate buffer solution, pH 8.5), and incubated for 30 min. After incubation, Fuc-MIP MNPs was washed with 50 mM phosphate buffer (pH 8.5) three times and then added with 50 μL 0.2 M ANTS aqueous solution and 50 μL 1.0 M $NaBH_3CN$ DMSO solution and incubated at 37°C in dark for 30 minutes. After that, Fuc-MIP MNPs were tightly washed with 50 mM phosphate buffer (pH 8.5) three times to remove the excess labeling reagent. Then, Fuc-MIP and NIP MNPs were resuspended and eluted in 50 μL, 0.1 M HAc for 2 h on a rotator. The MNPs were magnetically separated and the eluates were collected. Finally, the fluorescence of the supernatant in each tube was read on the microplate reader and the fluorescence intensity at excitation 356 nm and emission 512 nm was blank-subtracted and averaged over each tube.

3.15 The selectivity of LCA toward monosaccharides

Boronate affinity sandwich assay[7] was used to investigate the selectivity of lectin toward different monosaccharides. A boronic acid-functionalized 96-well microplate was used for the experiments. To modify the inner surface of a 96-well microplate with boronic acid, amino group was first introduced onto the surface of the microplate. The wells were filled with a 3: 1

(v/v) H_2SO_4 (98%)/HNO_3 (63%) mixture (250 μL/well) and kept at room temperature for 30 min. After being washed with water several times to achieve a neutral pH, the wells were filled with 5% aqueous APTES solution, pH 6.9 (250 μL/well), and slightly shaken at room temperature for 2 h then washed with anhydrous methanol and dried by air. After the wells were filled with 5.0 mg/mL FPBA dissolved in anhydrous methanol (250 μL/well), the microplate was sealed and slightly shaken at room temperature for 12 h. Subsequently, each well was supplemented with 100 μL of 1.0 mg/mL $NaBH_3CN$ dissolved in anhydrous methanol, and then, the microplate was sealed again and gently shaken at room temperature for 24 h. Finally, the solutions in the wells were disposed, and the wells were washed with anhydrous methanol 5-10 times and dried by air, then kept at 4°C for later experiments.

To immobilize Fuc on the inner surface of the boronic acid-functionalized 96-well microplate, 250 μL Fuc solution (1.0 mg/mL, pH 7.4, 10 mM phosphate buffer) was added into the different wells separately and incubated for 2 h at 25°C, then the 96-well microplate was rinsed with phosphate buffer (pH 7.4, 10 mM) three times. After that, 250 μL LCA-FITC (0.2 mg/mL, pH 7.4) was added into the immobilized-monosaccharide wells and incubated at room temperature in dark for 2 h, then the 96-well microplate was gently rinsed with 10 mM phosphate buffer (pH 7.4) three times. After that, 50 μL of 0.1 M HAc solution was added for elution, and the fluorescence intensity of the eluent was detected at excitation 485 nm and emission 528 nm.

3.16 Adsorption isotherm and binding constant measurement of Fuc-imprinted MNPs

Using MNPs as the substrate for preparation Fuc-imprinted NPs also for easy magnetic separation. Due to the poor UV absorbance of monosaccharides, the measurement of the K_d value for Fuc-imprinted MNPs was carried out in a special way. Equivalent Fuc-imprinted MNPs and non-imprinted MNPs (20 mg each) were added to 0.4 mL of different concentrations of Fuc solution in 0.5-mL centrifugal tubes, respectively. The tubes were shaken for 30 min at

room temperature. The MNPs were magnetically collected and rinsed with 0.5 mL of phosphate buffer (20 mM, pH 8.5) three times. After washing, the MNPs were resuspended in 50 μL ANTS (0.2 M, solvent H_2O) and 50 μL $NaBH_3CN$ (1.0 M, solvent: DMSO) for more than 30 min at 37°C in dark. To remove excess labeling reagents, the MNPs were magnetically collected and rinsed with 0.5 mL of phosphate buffer (20 mM, pH 8.5). After washing, the MNPs were resuspended and eluted in 50 μL 0.1 M HAc. Finally, the MNPs were magnetically separated and the eluates were collected. The amount of Fuc captured by the imprinted and non-imprinted MNPs were determined by measuring the ANTS-labeled monosaccharide in the eluates according to the fluorescence intensity at excitation 356 nm and emission 512 nm, which were plotted against the concentration of the Fuc solutions to obtain the adsorption isotherm. The Hill equation was used to fit the data to obtain the dissociation constant (K_d) of Fuc-imprinted MNPs toward Fuc.

3.17 Affinity capillary electrophoresis

To investigate the interaction between LCA lectin and Fuc, affinity capillary electrophoresis (ACE) assay was performed according to a previous report.[8] The running buffer was prepared with 50 mM phosphate buffer (pH 7.4) and varying concentrations of Fuc. And the sample was a mixture contained 0.02% (v/v) DMSO (electro-osmotic flow marker) and LCA (200 μg/mL). The electropherogram was recorded by UV absorbance detector at 214 nm. The viscosity correction factor was determined from separate viscosity measurements by measuring the migration time of a plug of 0.02% (v/v) DMSO, under a certain pressure, within the same capillary filled with the running buffer under investigation. The differences of mobility between lectin-monosaccharide complex and lectin are calculated according to retention time changes based on different monosaccharide concentration contained in the background buffer. The K_d value was calculated by fitting the differences of mobility changes according to the different concentration monosaccharides.

3.18 Determination of AFP and AFP-L3 in human serum by triMIP-PISA

Serum samples from 10 HCC patients and 4 healthy individuals were determined by the triMIP-PISA method developed in this study. Some serum samples of patients were diluted with PBS buffer, to reduce the Raman signal intensity to the linear range of the calibration curves. The dilution folds for the patient samples P1, P3, P4, P7, P8, and P10 are 100, 100, 100, 5, 100, and 20, respectively.

A 5-μL volume of serum samples was dropped on each spot of an N-terminal epitope-imprinted extraction array and allowed extraction for 15 min in a humidity chamber, followed by rinsing with 10 mM phosphate buffer (pH 7.4) three times. Captured protein was separately labeled with 5 μL of C-terminal epitope-imprinted Ag/PATP@SiO_2 NPs and Fuc-imprinted Ag/NTP@SiO_2 NPs for 15 min in a humidity chamber. The spots were washed with phosphate buffer (10 mM, pH 7.4) three times, dried at room temperature and then detected the Raman spectrograph.

3.19 Determination of AFP and AFP-L3 in human serum by immunofluorescence assay

LCA-based immunofluorescence assay (LCA-IFA) of the same set of samples was carried out at the independent clinical laboratory at Medical testing laboratory (Shandong, China). Briefly, a sample was mixed with an AFP-specific antibody conjugated to a fluorescent label for detection. The mixture was then loaded into the microchannel, reacted with monoclonal antibody carrying a mobility modifier, and stacked by isotachophoresis to form AFP immunocomplexes. High voltage was applied across the isotachophoresis channel during the isotachophoresis process. The cathode was switched to the handoff position after isotachophoresis stackinag was completed, and immunocomplexes containing AFP-L3 were separated from those containing AFP-L1 through binding to LCA in the separation channel during the chip gel electrophoresis phase. Size separation between these two populations of

immunocomplex leads to the resolution of both isoforms. The quantification of AFP-L3 and AFP-L1 was achieved according to the areas of the separated bands.

References

1 L. Zhou, Y. Wang, R. Xing, J. Chen, J. Liu, W. Li and Z. Liu, *Biosens. Bioelectron.* 2019, **145**, 111729.

2 R. Xing, Z. Guo, H. Lu, Q. Zhang and Z. Liu, *Sci. Bull.* 2022, **67**, 278.

3 R. Xing, Y. Wen, Y. Dong, Y. Wang, Q. Zhang and Z. Liu, *Anal. Chem.* 2019, **91**, 9993.

4 R. Xing, S. Wang, Z. Bie, H. He and Z. Liu, *Nat. Protoc.* 2017, **12**, 964.

5 S. Wang, D. Yin, W. Wang, X. Shen, J.-J. Zhu, H.-Y. Chen and Z. Liu, *Sci. Rep.* 2016, **6**, 22757.

6 Z. Bie, Y. Chen, J. Ye, S. Wang and Z. Liu, *Angew. Chem. Int. Edit.* 2015, **54**, 10211.

7 X. Bi and Z. Liu, *Anal. Chem.* 2014, **86**, 12382.

8 C. Lu, H. Li, H. Wang and Z. Liu, *Anal. Chem.* 2013, **85**, 2361.

Supplementary Data

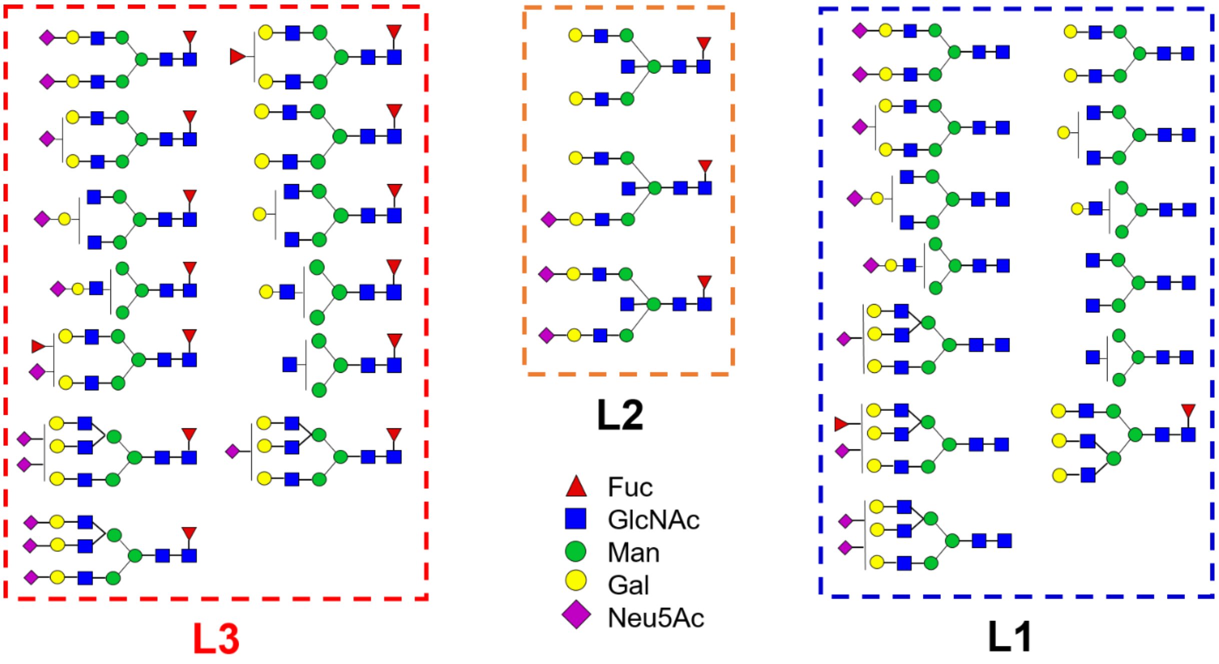

Fig. S1 Classification of the possible glycans of alpha-fetoprotein. The glycans of AFP can be classified into three classes, i.e., L1, L2 and L3, based on their reactivity to LCA on affinity electrophoresis. Class L1 does not react with LCA. The L1 fraction of the total AFP is present in chronic hepatitis and liver cirrhosis, and constitutes a majority fraction of total AFP in the non-malignant liver diseases. Class L3 has LCA-binding activity, and has an additional α1-6 fucose residue attaching at terminus of N-acetylglucosamine. It appears to be produced only by cancerous cells. Class L2 showed an intermediate affinity to LCA. AFP-L2 is mostly derived from yolk sac tumours and also could be detected in maternal serum during pregnancy.

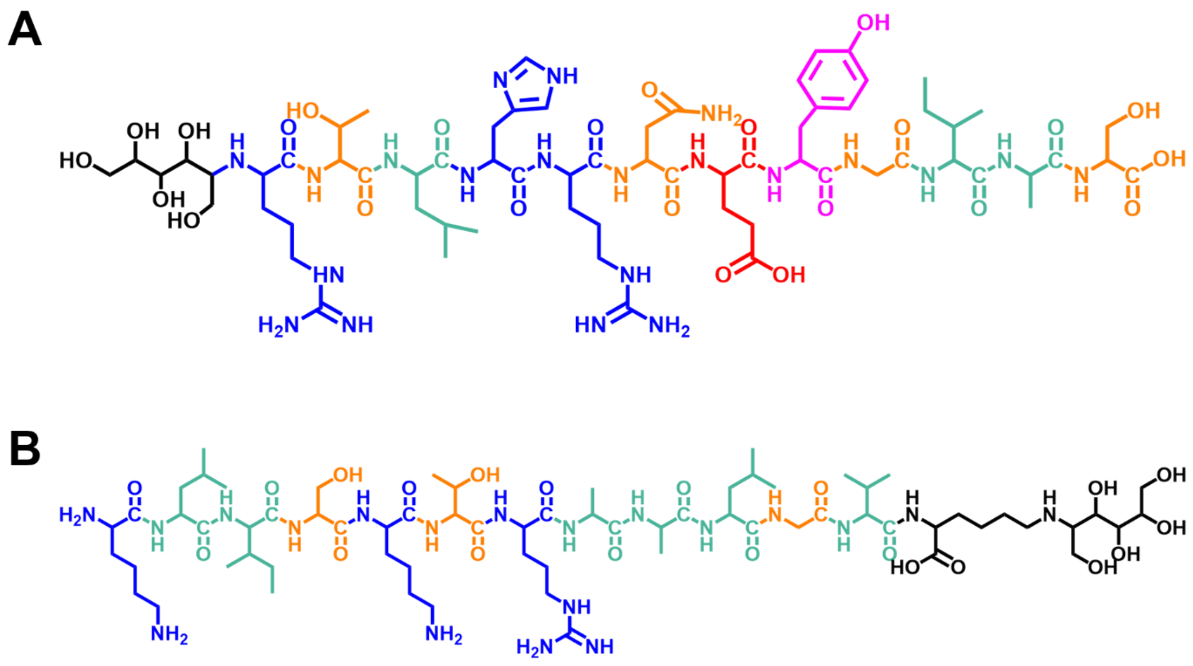

Fig. S2 The structure of (A) glycated N-terminal epitope (Fru-RTLHRNEYGIAS) and (B) glycated C-terminal epitope (KLISKTRAALGVK-Fru) of AFP.

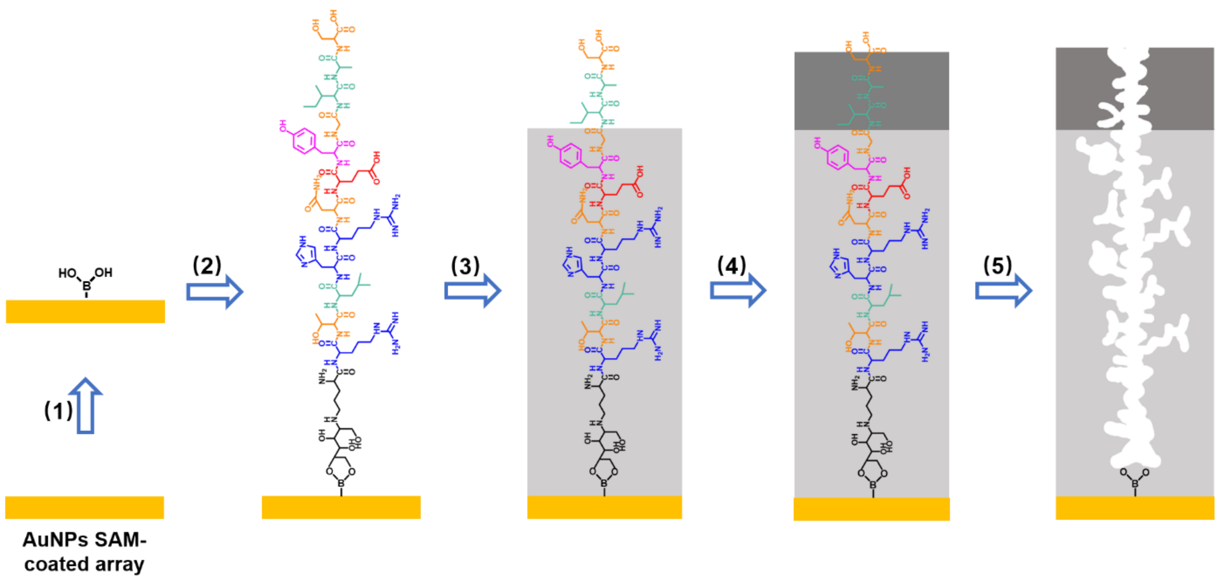

Fig. S3 Synthesis route of AFP N-terminal epitope-imprinted array. Steps: (1) Boronic acid-functionalization, (2) Immobilization, (3) Imprinting, (4) Cladding, (5) Template removal. Gray area: the imprinting layer, Dark gray area: the cladding layer.

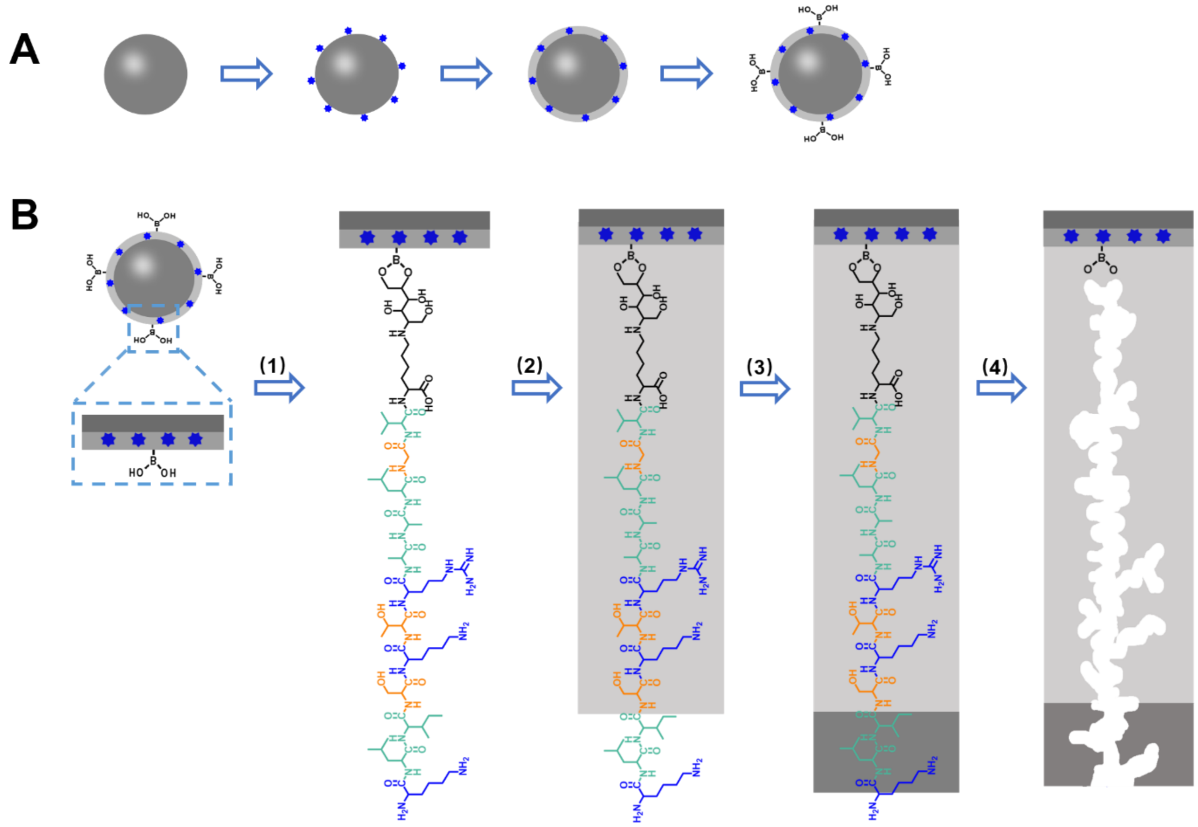

Fig. S4 Synthesis route of (A) boronate functionalized SERS nanotags and (B) AFP C-terminal epitope-imprinted SERS nanotags. Steps in B: (1) Immobilization, (2) Imprinting, (3) Cladding, (4) Template removal. Gray area: the imprinting layer, Dark gray area: the cladding layer.

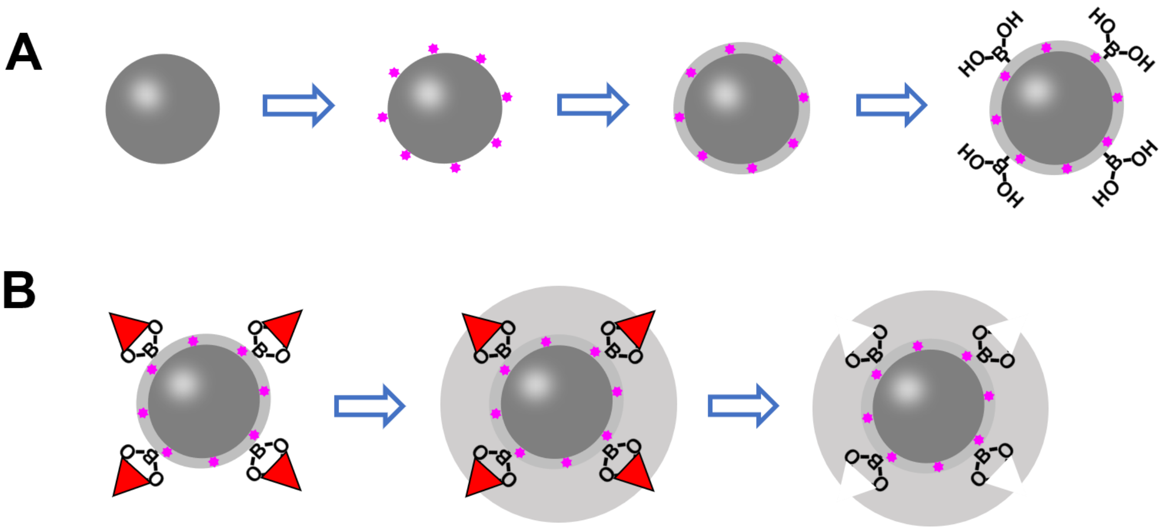

Fig. S5 Synthesis route of (A) boronate functionalized SERS nanotags and (B) Fuc-imprinted SERS nanotags (red triangle: Fuc template, the light gray area: the imprinting layer).

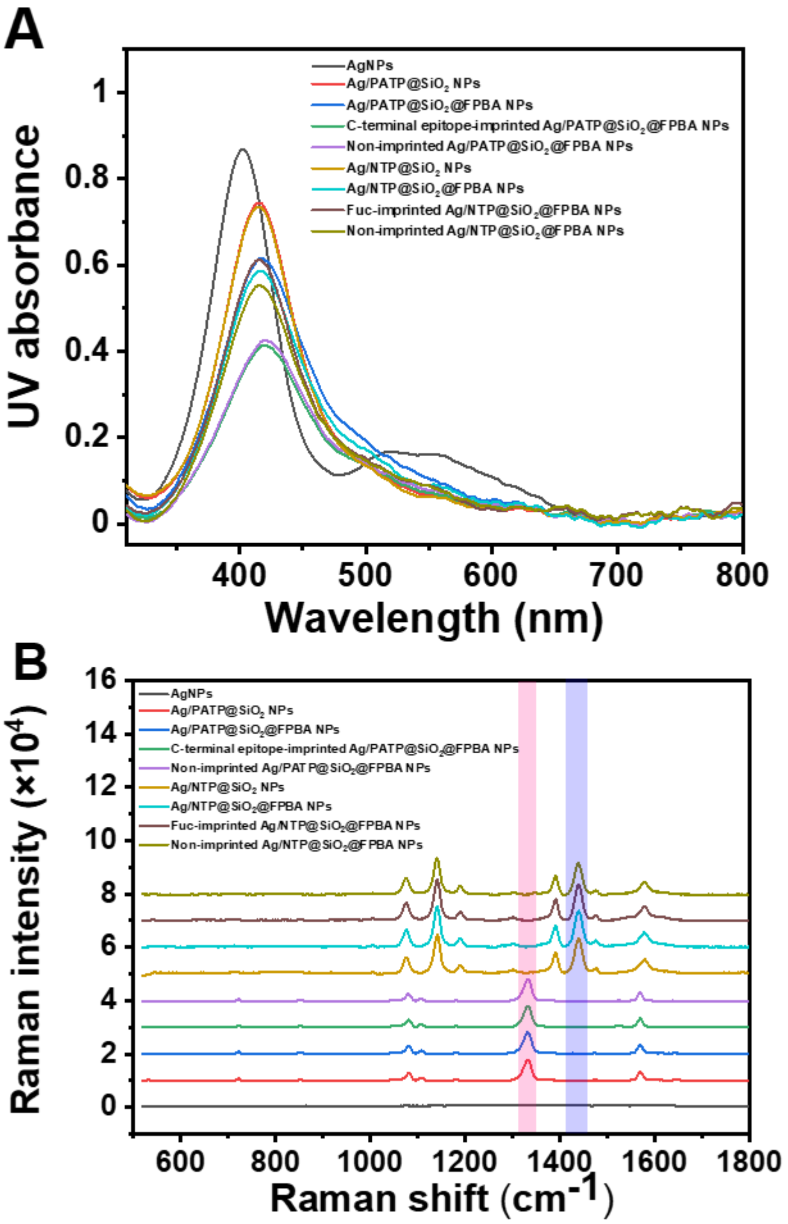

Fig. S6 (A) UV-vis spectra of AgNPs, Ag/PATP@SiO_2 NPs, Ag/PATP@SiO_2@FPBA NPs, C-terminal epitope-imprinted and non-imprinted Ag/PATP@SiO_2@FPBA NPs, Ag/NTP@SiO_2 NPs, Ag/NTP@SiO_2@FPBA NPs, Fuc-imprinted and non-imprinted Ag/NTP@SiO_2@FPBA NPs. (B) Raman spectra of AgNPs, Ag/PATP@SiO_2 NPs, Ag/PATP@SiO_2@FPBA NPs, C-terminal epitope-imprinted and non-imprinted Ag/PATP@SiO_2@FPBA NPs, Ag/NTP@SiO_2 NPs, Ag/NTP@SiO_2@FPBA NPs, Fuc-imprinted and non-imprinted Ag/NTP@SiO_2@FPBA NPs.

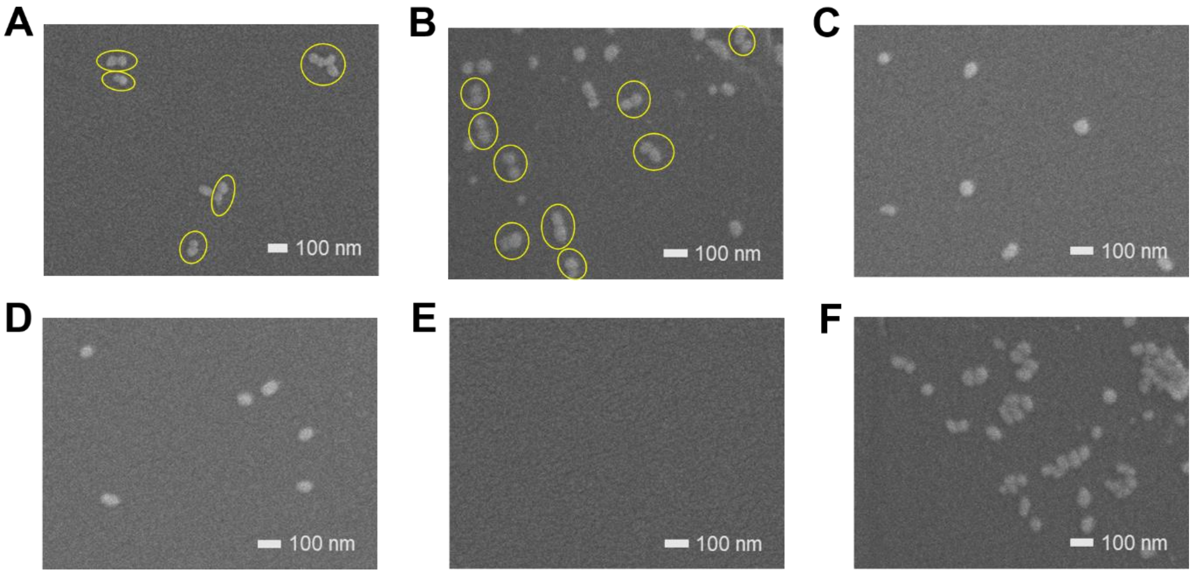

Fig. S7 SEM characterization of the labeling of AFP and its fucosylation on AFP N-terminal epitope-imprinted array. (A) and (B) Simultaneous labeling of AFP and its fucosylation by AFP C-terminal epitope-imprinted nanotags and Fuc-imprinted nanotags, respectively. (C) Labeling of AFP by AFP C-terminal epitope-imprinted nanotags. (D) Labeling of AFP fucosylated glycoform by Fuc-imprinted nanotags. (E) Simultaneous labeling of a blank sample by the two types of nanotags. (F) Simultaneous labeling of AFP and its fucosylation by the two types of nanotags without removal of unbound nanotags. AFP concentration: except that 2 ng/mL was used for (B) and blank sample was used for (E), the concentration for (A), (C), (D) and (F) was 1 ng/mL. Other conditions were the same as those used for subsection 3.18.

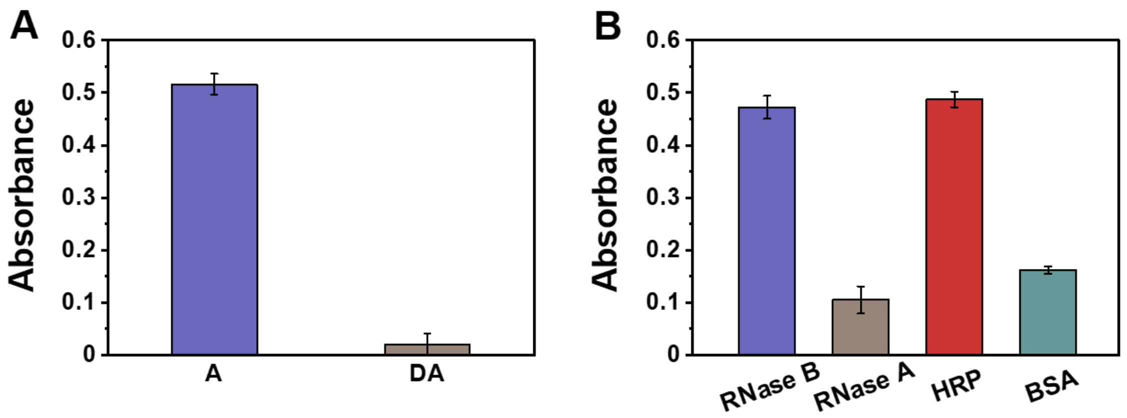

Fig. S8 Comparison of the amounts of different compounds captured by Fe_3O_4@SiO_2@DFFPBA MNPs. Samples: (A) adenosine (A) and deoxyadenosine (DA) (1.0 mg/mL each), and (B) RNase A, RNase B, HRP or BSA (1.0 mg/mL each) dissolved in the binding buffer. Binding buffer: ammonium bicarbonate (50 mM, pH 8.5) containing 150 mM NaCl; Elution solution: 100 mM HAc. The error bars represent the standard deviation for three parallel experiments.

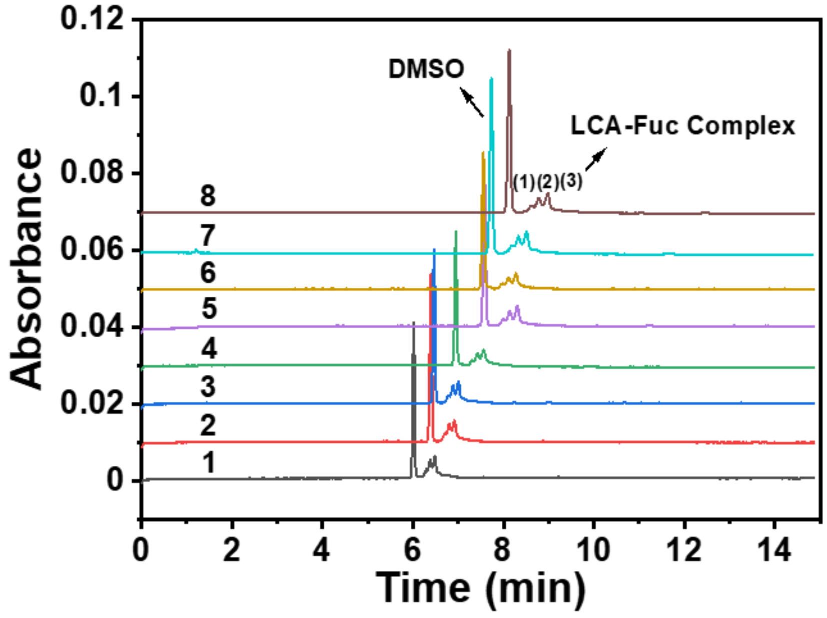

Fig. S9 Electropherograms for LCA interacting with Fuc of different concentrations. Concentration of Fuc added in the background buffer, from 1 to 8: 0, 0.1, 0.2, 0.5, 0.8, 1.0, 2.0, and 5.0 mg/mL. Background buffer: 50 mM phosphate buffer (pH 7.4). Peaks (1) - (3): different isoforms of LCA-Fuc complex.

Table S1 Comparison of the measured concentrations of AFP and AFP-L3 and their ratios for different samples by triMIP-PISA and LCA-IFA.

Sample [*]	triMIP-PISA			LCA-IFA		
	AFP (ng/mL)	AFP-L3 (ng/mL)	Ratio (%)	AFP (ng/mL)	AFP-L3 (ng/mL)	Ratio (%)
P1	481.9 ± 16.5	252.3 ± 30.4	52.5 ± 7.5	454.3	318.5	70.1
P2	9.7 ± 3.2	2.5 ± 0.5	26.3 ± 3.6	3.7	1.2	31.2
P3	527.9 ± 31.0	273.6 ± 9.0	52.0 ± 3.7	538.7	286.6	53.2
P4	1155.0 ± 136.1	286.0 ± 60.3	24.6 ± 3.4	1826.5	484.0	26.5
P5	8.0 ± 0.3	3.5 ± 0.3	44.1 ± 3.2	6.6	2.1	32.1
P6	9.5 ± 1.5	2.8 ± 0.2	30.1 ± 2.2	6.2	2.0	32.4
P7	58.6 ± 3.0	7.9 ± 0.9	13.4 ± 1.7	63.7	8.8	13.8
P8	1152.3 ± 425.1	266.4 ± 80.9	23.5 ± 0.14	1926.9	713.0	37.0
P9	8.9 ± 2.6	3.1 ± 1.2	33.8 ± 3.4	4.5	1.4	31.4
P10	237.5 ± 20.9	66.2 ± 9.3	27.9 ± 3.1	249.2	71.0	28.5
H1	5.45 ± 0.74	0.10 ± 0.02	1.8 ± 0.2	6.6	0.4	6.1
H2	4.46 ± 1.30	0.13 ± 0.02	2.9 ± 0.5	6.6	0.4	6.1
H3	3.57 ± 0.42	0.14 ± 0.03	4.0 ± 0.8	4.5	0.3	6.7
H4	2.56 ± 0.49	0.13 ± 0.05	5.1 ± 0.1	3.4	0.0	0.0

[*] P: patient of HCC, H: healthy individual.
